# Volatile Composition of Smoked and Non-Smoked Iranian Rice

**DOI:** 10.3390/foods5040081

**Published:** 2016-11-30

**Authors:** Leontina Lipan, Mohammad Hojjati, Hussein El-Zaeddi, Lucía Sánchez-Rodríguez, Ángel Antonio Carbonell-Barrachina

**Affiliations:** 1 Department of Agro-Food Technology, Miguel Hernández University of Elche, Carretera Beniel, km 3.2, 03312 Orihuela, Alicante, Spain; leontina.lipan@yahoo.com (L.L.); ElZaeddi@gmail.com (H.E.-Z.); lucia.sanchez@goumh.umh.es (L.S.-R.); 2 Department of Food Science and Technology, Ramin Agriculture and Natural Resources University of Khuzestan, Mollasani 63417-73637, Ahvaz, Iran; hojjatim@yahoo.com

**Keywords:** aldehydes, furans, GC-MS, HS-SPME, *Oryza sativa*, phenol derivatives

## Abstract

In this work, the volatile profiles of smoked and non-smoked Iranian rice were identified, and their relative abundance was calculated and compared. Headspace solid-phase microextraction together with gas chromatography-mass spectrometry (SPME-GC-MS) were used to extract and identify the volatile compounds. The main groups of volatiles in Iranian rice were aldehydes, ketones, phenol derivatives, furans, linear hydrocarbons, esters and terpenes. The chemical family aldehydes was the most abundant one in the profile of non-smoked rice, while phenol derivatives and furans predominated in smoked samples. This study is the first one reporting comparative data of volatile compounds between smoked and non-smoked Iranian rice.

## 1. Introduction

Rice (*Oryza sativa*) is an essential food for the people of the world, being the second major crop after wheat [1]. Iran is also a relevant world rice producer and exporter [2]. The most important area of rice cultivation, with more than 80% of rice production, is the north part of Iran and includes the provinces of Mazandaran and Gilan, with 238,000 ha planted area [1,2,3]. Even the local rice varieties have low grain yields (averaging 2.5 to 3.5 tones/ha), and more than 80% of the total rice area in Iran is still under these traditional varieties because of their fragrance and other excellent quality traits [3,4].

After the harvest, some rice cultivars undergo a smoking process to enhance the flavor. Exposing bulk rice filled in cotton bags to the smoke obtained by burning special woods, such as those from beech, alder, oak, and fruit trees, for several hours is the traditional method for flavoring and preserving rice. Smoked rice consumption is common in many parts of Iran, especially in northern areas.

The fragrance and taste of the Iranian aromatic rice are the main reasons supporting the higher prices for this type of rice as compared to non-aromatic cultivars in Iran [3]. The compounds responsible for this high quality of the aromatic rice samples are the volatile compounds released from the grains [5]. They have been fully investigated, due to their important aspects for consumer preference and acceptance [6]. For instance, it has been demonstrated that 2-acetyl-1-pyrroline (2-AP) is a volatile compound with an attractive aroma, the sensory descriptors of which are: popcorn, pandan (*Pandanus amaryllifolius*), nutty, and white bread. Also, the 2-AP can be detected in non-aromatic rice, but at trace levels [6,7].

More than 200 volatiles have been identified in rice using the traditional methods which include static headspace, purge and trap, steam distillation-solvent extraction, and direct solvent extraction for collection/concentration [8]. The separation is conducted by gas chromatography with flame ionization or a mass spectrometer [9]. Static headspace (SHS) is an effective technique for simple and rapid quantitative analysis of the aroma compound of rice, in which the sample headspace is automatically brought directly to the gas chromatograph (GC) [10]. However, Ezquerro et al. demonstrated that headspace solid-phase microextraction (HS-SPME) is more sensitive than SHS for the identification of volatile compounds from the packaged materials [11]. Also, Lin et al. investigated the volatile compounds of rice using SPME combined with gas chromatography and mass spectrometry (GC-MS); this method proved to have several advantages, including easy preparation, quick analysis, no use of organic solvents and low cost [12]. The extraction temperature and exposure time are the most important factors in these techniques [10,11].

“Hashemi” is the most important aromatic rice cultivar in Iran [3]; also “Sadri” is considered a rice genotype with a strong aroma [13]. Moreover, there are studies about the “Domsiah” aromatic rice cultivar, where the main volatile compounds were nonanal (18.2%), hexanal (14.3%), 2-benzoyl-6,7-dimethoxy-4-methylidene-2H-1,3-benzothiazine (7.8%), epilophodione (7.0%), and 1-methyl-4-(1-methylethenyl)cyclohexene (6.6%) [14]. In general, the volatile compounds that have been identified in rice are alcohols, aldehydes, ketones, esters, hydrocarbons, heterocyclic compounds and organic acids, with the predominant family being aldehydes [12].

Even though there are several studies about the volatile profiles of rice, there is scarce information on the aromatic components of smoked rice. Consequently, the purpose of this study was to isolate, identify, and compare the volatile profiles of smoked and non-smoked rice samples from different Iranian cultivars. Fifteen rice cultivars four of which were smoked, were evaluated by HS-SPME combined with GC-MS.

## 2. Materials and Methods

### 2.1. Experimental Material

Fifteen rice samples were cultivated in non-organic farms from various provinces of Iran and were harvested in summer of 2012. Most of the samples were grown in the north part of Iran (Table 1), and the number of samples to be harvested and analyzed was decided according to the rice productions of the different regions. Unfortunately, it was not possible to get similar numbers of samples from non-smoked and smoked rice, but the ratio of samples non-smoked:smoked (11:4) is quite representative of the one reaching the Iranian market.

The products were obtained by Department of Food Science and Technology of Ramin Agriculture and Natural Resources University, Iran. Samples were obtained in local markets, to fully reproduce the type of samples being marketed in Iran and available in the main Iranian cities. Samples were bought in triplicate, meaning samples from three different batches were obtained and used for this study. Just after collection, the rice samples were immediately air-mailed to the Universidad Miguel Hernández de Elche (Spain) where they were analyzed for their volatile profiles.

Food smoking is an old process of flavoring, cooking, and preserving foods using wood smoke. Exposing cotton bags of rice (up to 50 kg) to the smoke of burning special forest woods, such as beech, alder, oak, and fruit-tree for several hours (4–5 h) is the traditional method of its flavoring and preserving in Iran and it is called in Persian "berenge doudi". The process is done in a wooden cottage it is quite similar in all areas of northern Iran. However, in future studies the effect of the different parameters involved in the smoking process will be studied, for instance rice variety, type of wood, time of smoking, etc.

### 2.2. Extraction Method

Headspace solid phase micro-extraction (HS-SPME) was used to isolate the volatile compounds from the rice samples. Approximately 5 g of ground rice and 10 mL of ultrapure water was settled in 50 mL vial with polypropylene cap and PTFE/silicone septa. Moreover, 0.75 g NaCl, 1 µL anethole (1 mg/L, used as internal standard), and a magnetic stirring bar were added to the vial. The vial was settled in a 40 °C water bath and kept for 15 min to reach an equilibrium between the rice sample mixed in water and the headspace. After this equilibration time was elapsed, a 50/30 µm DVB/CAR/PDMS (divinylbenzene/carboxen/polydimethylsiloxane) fiber (StableFlex/SS, 2 cm, and 24 Ga; Supelco, Sigma-Aldrich Co, Bellefonte, PA, USA) was introduced into the vial through a hole in the cap and exposed to the sample headspace for 50 min at 40 °C (simulating approximately the mouth temperature). After the extraction process, the fiber was quickly removed and injected in the port of gas chromatograph and kept for 3 min in the desorption port. This type of fiber was adequate for its high capacity to trap volatile chemicals, and provided excellent results in other matrixes, such as pistachios or pomegranate juice [15,16].

### 2.3. Chromatographic Analyses

The rice volatile compounds were analyzed and identified using a Shimadzu GC-17A gas chromatograph connected with a Shimadzu QP-5050A mass spectrometer detector (Shimadzu Corporation, Kyoto, Japan). The GC-MS system consisted of a TRACSIL Meta.X5 (95% dimethylpolysiloxane and 5% diphenylpolysiloxane) column (60 m × 0.25 mm, 0.25 µm film thickness; Teknokroma S. Coop. C. Ltd, Barcelona, Spain). The carrier gas used to perform the analysis were helium, at a column flow rate of 0.6 mL·min^−1^, and a total flow of 6.0 mL·min^−1^ in a split ratio of 1:6. The oven program was as follows: (a) 80 °C for 0 min; (b) increase of 3 °C·min^−1^ from 80 °C to 210 °C, and hold for 1 min; (c) increase of 25 °C·min^−1^ from 210 °C to 300 °C, and hold for 3 min. The injector and detector temperatures were 230 °C and 300 °C, respectively.

Three protocols were used to identify the rice volatile compounds: (1) retention indices and its correlation with those from the literature; (2) GC-MS retention times (authentic compounds); (3) mass spectra (authentic chemicals and NIST05 spectral library collection) [17]. Only fully identified compounds have been reported in this study.

The relative abundance of the volatile compounds (%) was performed on a gas chromatograph, Shimadzu 2010, with a flame ionization detector (FID). The column and chromatographic conditions were those previously reported for the GC-MS analysis. The injector temperature was 200 °C and nitrogen was used as carrier gas (1 mL·min^−1^). The relative abundance was obtained from electronic integration measurements using flame ionization detection (FID). Anethole (1000 mg·L^−1^) was added as internal standard at the beginning of the distillation procedure to simulate the behavior of volatile compounds; this chemical was used as internal standard after checking that it was absent in herbs, it separates well from other volatiles, it possesses similar FID and MS response factors to most of the volatiles in the aromatic herb essential oil, it is stable at high temperatures and does not react with water. This internal standard, anethole, was used to normalize the area of all the rice volatiles.

The volatile composition analysis was run in triplicate and results were expressed as a percentage of the total area represented by each one of the volatile compounds [16,18]. Thus, GC-MS was used for identification of the volatile compounds while GC-FID was used for establishing the relative abundance of the volatiles. This combination of GC-MS and GC-FID has been successfully used for the identification and relative abundance (%) or quantification (μg·L^−1^ or μg·kg^−1^) of volatile compounds in several matrices, such as aromatic herbs [18,19], wild bitter almonds (*Amygdalus scoparia*) [20], and *Origanum majorana* [21], among others.

### 2.4. Statistical Analysis

To compare the data two consecutive tests were performed: (i) one-way analysis of variance (ANOVA), and (ii) Tukey’s multiple-range test. To be considered statistically significant the differences must be, of at least, at *p* < 0.05. All statistical analyses were performed using StatGraphics Plus 5.0 software (Manugistics, Inc., Rockville, MD, USA).

## 3. Results

### 3.1. Identification of Volatile Compounds in Rice

Table 2 shows the 37 volatile compounds that have been identified by GC-MS. These compounds can be grouped into seven main chemical families: (i) aldehydes (10 compounds); (ii) ketones (four); (iii) phenol derivatives (11); (iv) furans (eight); (v) linear hydrocarbons (two); (vi) esters (one); and (vii) terpenes (one). Table 2 also shows the main sensory descriptors of each of the volatiles identified in the rice profile. From this list, it is important to highlight that several compounds have descriptors related to the wood fumes (smoked notes), such as furfural, 2-acetylfuran, 5-methyl-furfural, benzofuran, and guaiacol.

### 3.2. Volatile Compositions of Non-Smoked and Smoked Iranian Rice

The volatile profile of non-smoked rice was dominated by only five compounds: tetradecane (32.9%), hexanal (17.6%), benzaldehyde (14.0%), *p*-ethylguaiacol (13.7%), and nonanal (9.9%) (Table 3); the concentrations of these compounds were higher than those in the non-smoked samples. Most of the compounds found in non-smoked rice samples were already found in California long-grain rice by Buttery et al. [23], as early as1988, and later confirmed in other studies [24,25]. In this way, Malekzadeh et al. also obtained similar results by concluding that the major compounds identified in Iranian rice were alkyl aldehydes [10].

However, the application of smoking as a post-harvest unit operation significantly increased the concentrations of several compounds, including furfural (26.7%), guaiacol (11.3%), phenol (8.1%), 2-methoxy-4-methylphenol (7.0%), *p*-cresol (5.4%), 5-methyl-furfural (4.4%), and 2-acetylfuran (1.9%). These results agreed quite well with the data provided by Pino [26], who reported that the main compounds (concentrations above 5% GC area) in the smoke flavoring from rice husk were: 2-furfural, phenol, 2-methoxyphenol, 4-ethyl-2-methoxyphenol, and 2,3-dimethoxyphenol.

Table 4 shows the total contents (%) of the main chemical families found in non-smoked and smoked rice samples. Consequently, it can be concluded that the volatile profiles of non-smoked rice were controlled by aldehydes (46.5%) and linear hydrocarbons (33.3%), while those of smoked samples were dominated by phenol derivatives (44.9%) and furans (35.6%).

### 3.3. Principal Component Analysis (PCA)

**Figure 1:**
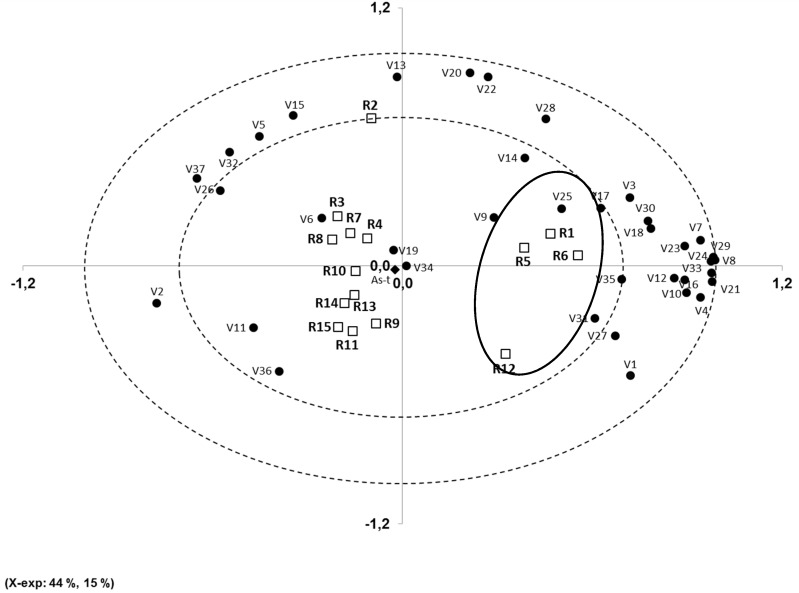
PCA scores plot showing relationship among volatile compounds and rice sample (● volatile compound, □ rice sample).

The PCA scores plot (Figure 1) successfully group the four smoked rice samples (R1, R5, R6, and R12). Besides, it can be observed that around these four samples, most of the compounds found belonged to two chemical families, phenol derivatives and furans; this result agrees quite well with the previous comments and results compiled in Table 4. The aldehyde and linear hydrocarbon groups were related to the 11 remaining samples of non-smoked rice and are located on the left side of the graph.

The main compounds related to the volatile profiles of non-smoked rice were V6 (heptanal), V11 (benzaldehyde), V19 (limonene), V34 (2-decenal), and V36 (p-ethylguaiacol), among others, while smoked samples were positively grouped with compounds such as V9 (anisole), V25 (methylbenzoate), V31 (2-methoxy-4-methylphenol), V27 (2-ethylphenol), V17 (2-propionylfuran), and V3 (3-furaldehyde), among others. These graphical results fully agree with data included in Table 3 and Table 4 and previously discussed.

The rice sample R2 (“Domsiah”) presented a very different profile, with the volatiles V13 (6-methyl-5-hepten-2-one), V15 (octanal), V20 (3-octen-2-one), and V22 (2-octenal), among others, playing a differential role. The volatiles V6 (heptanal), V11 (benzaldehyde), V19 (limonene), V34 (2-decenal), and V36 (p-ethylguaiacol) were those common to all non-smoked samples.

## 4. Conclusions

The volatile profiles of non-smoked rice were controlled by aldehydes (hexanal, benzaldehyde, nonanal, and decanal) and linear hydrocarbons (tetradecane), while those of smoked samples were dominated by phenol derivatives (guaiacol, phenol, 2-methoxy-4-methylphenol, etc.) and furans (furfural, and 5-methylfurfural). In the future, it will be necessary to conduct consumer studies to be able to establish correlations among the volatile compounds and the consumer preferences.

## Figures and Tables

**Table 1:** Specifications of the samples of Iranian rice.

Sample Code	Cultivar	Comments	Province	City	Location
R1	Sadri	Smoked	Guilan	Astaneh	North
R2	Domsiah	-	Guilan	Astaneh	North
R3	Shiroudi	-	Mazandaran	Amol	North
R4	Hashemi	-	Guilan	Astaneh	North
R5	Sadri	Smoked	Guilan	Rasht	North
R6	Sadri	Smoked	Guilan	Talesh	North
R7	Hashemi	-	Mazandaran	Mahmudabad	North
R8	Tarem	-	Mazandaran	Mahmudabad	North
R9	Hashemi	-	Mazandaran	Fereydun kenar	North
R10	Tarem	-	Mazandaran	Amol	North
R11	Champa	-	Khouzestan	Ramhormoz	West-South
R12	Tarem	Smoked	Mazandaran	Fereydun kenar	North
R13	Lenjun	-	Isfahan	Lenjan	Center
R14	Tarem	-	Lorestan	Borujerd	West
R15	Shamshiri	-	Ilam	Chardaval	West

**Table 2:** Identification of volatile compounds, by GC-MS, in smoked and non-smoked Iranian rice.

Compound	Sample Code	RT ^†^ (min)	I^T ‡^		Odor Descriptor ^‡^
			Exp ^‡^	Lit ^†^	
2-Methylfuran	V1	5.55	600	605	Ethereal, acetone, chocolate
Hexanal	V2	7.39	804	801	Fatty, green
3-Furaldehyde	V3	7.69	820	831	
Furfural	V4	8.07	839	830	Almond, woody
2-Heptanone	V5	9.18	895	891	Banana, cinnamon, spicy, fruity
Heptanal	V6	9.50	908	903	Oily, fruity, woody, fatty, nutty
2-Methyl-2-cyclopenten-1-one	V7	9.80	918	915	
2-Acetylfuran	V8	9.87	920	921	Almond, caramel, coffee
Anisole	V9	10.10	928	926	Alcohol, butter, cheese, ethereal
5-Methyl-furfural	V10	11.43	972	978	Almond, caramel, spicy
Benzaldehyde	V11	11.71	982	978	Almond, cherry, sweet
Phenol	V12	11.77	984	980	Plastic
6-Methyl-5-hepten-2-one	V13	11.95	990	994	Oily, herbaceous, green
2-Amylfuran	V14	12.23	999	1001	Fruity, green, earth, bean
Octanal	V15	12.68	1011	1006	Honey, fruity, fatty, citrus
Benzofuran	V16	12.86	1016	1015	Burnt, coffee, woody
2-Propionylfuran	V17	13.02	1020	1024	
*p*-Methylanisole	V18	13.58	1034	1026	Floral, earthy, walnut
Limonene	V19	13.89	1042	1039	Herbaceous, minty
3-Octen-2-one	V20	14.03	1046	1040	Berry, nutty, earthy, vegetable
2-Methyl-phenol	V21	14.75	1065	1075	
2-Octenal	V22	14.89	1068	1060	Spicy, herbaceous, green
*p*-Cresol	V23	15.57	1086	1084	Medicinal
Guaiacol	V24	16.28	1104	1102	Woody, smoky
Methylbenzoate	V25	16.68	1113	1106	
Nonanal	V26	16.78	1115	1105	Fruity, citrus, grape, vegetable
2-Ethylphenol	V27	19.12	1168	1169	Oily, phenolic
2-Nonenal	V28	19.35	1173	1171	Waxy, fatty
4-Ethylphenol	V29	19.80	1183	1178	Alcohol, medicinal
3-Ethylphenol	V30	19.92	1186	1175	Musty, phenolic, burnt
2-Methoxy-4-methylphenol	V31	20.96	1209	1198	Almond
Decanal	V32	21.43	1219	1212	Waxy, floral, citrus, sweet
Cinnamaldehyde	V33	22.37	1239	1234	Cinnamon, clove, spicy
2-Decenal	V34	24.18	1278	1274	Floral, citrus, green, meaty
Tridecane	V35	24.78	1291	1300	
*p*-Ethylguaiacol	V36	25.00	1296	1290	Smoky, meat
Tetradecane	V37	25.26	1301	1290	Mild waxy

^†^ RT = retention time, I^T^ = linear retention indexes, Exp = experimental, and Lit = Literature; ^‡^ SAFC [22].

**Table 3:** Comparative composition of volatile compounds, by GC-FID, of smoked and non-smoked Iranian rice.

Compound	ANOVA ^†^	Non-Smoked Rice	Smoked Rice
		Relative Abundance (%)	
2-Methylfuran	*	0.29 ± 0.08 ^‡^ b ^¥^	0.41 ± 0.01 a
Hexanal	***	17.6 ± 2.5 a	2.50 ± 0.36 b
3-Furaldehyde	*	0.11 ± 0.02 b	0.26 ± 0.08 a
Furfural	***	0.14 ± 0.03 b	26.7 ± 2.4 a
2-Heptanone	**	0.49 ± 0.14 a	0.06 ± 0.02 b
Heptanal	*	0.46 ± 0.02 a	0.30 ± 0.08 b
2-Methyl-2-cyclopenten-1-one	*	0.06 ± 0.01 b	0.26 ± 0.07 a
2-Acetylfuran	***	0.07 ± 0.01 b	1.91 ± 0.24 a
Anisole	*	0.17 ± 0.07 b	0.29 ± 0.08 a
5-Methyl-furfural	***	0.22 ± 0.13 b	4.39 ± 0.50 a
Benzaldehyde	***	14.0 ± 2.9 a	2.69 ± 0.10 b
Phenol	***	0.22 ± 0.06 b	8.03 ± 0.14 a
6-Methyl-5-hepten-2-one	NS	0.45 ± 0.15	0.38 ± 0.09
2-Amylfuran	*	0.33 ± 0.11 b	1.03 ± 0.30 a
Octanal	*	0.91 ± 0.12 a	0.55 ± 0.10 b
Benzofuran	*	0.06 ± 0.01 b	0.66 ± 0.15 a
2-Propionylfuran	NS	0.06 ± 0.01	0.17 ± 0.09
*p*-Methylanisole	*	0.12 ± 0.04 b	0.61 ± 0.16 a
Limonene	*	0.60 ± 0.15 a	0.34 ± 0.06 b
3-Octen-2-one	NS	0.18 ± 0.05	0.23 ± 0.07
2-Methyl-phenol	***	0.11 ± 0.04 b	2.76 ± 0.09 a
2-Octenal	*	0.29 ± 0.11 b	0.50 ± 0.08 a
*p*-Cresol	***	0.07 ± 0.02 b	5.44 ± 0.83 a
Guaiacol	***	0.35 ± 0.17 b	11.3 ± 1.1 a
Methylbenzoate	*	0.12 ± 0.06 b	0.41 ± 0.16 a
Nonanal	***	9.89 ± 1.30 a	3.33 ± 0.45 b
2-Ethylphenol	***	0.06 ± 0.02 b	1.44 ± 0.34 a
2-Nonenal	*	0.15 ± 0.06 b	0.43 ± 0.12 a
4-Ethylphenol	***	0.05 ± 0.01 b	3.48 ± 0.84 a
3-Ethylphenol	**	0.08 ± 0.02 b	0.84 ± 0.39 a
2-Methoxy-4-methylphenol	***	2.06 ± 0.48 b	6.99 ± 0.06 a
Decanal	***	2.33 ± 0.44 a	0.45 ± 0.16 b
Cinnamaldehyde	*	0.13 ± 0.03 b	0.51 ± 0.06 a
2-Decenal	NS	0.84 ± 0.40	0.82 ± 0.36
Tridecane	**	0.35 ± 0.09 b	1.74 ± 0.47 a
*p*-Ethylguaiacol	***	13.7 ± 4.0 a	3.74 ± 1.20 b
Tetradecane	***	32.9 ± 4.9 a	4.12 ± 1.35 b

^†^ NS = not significant F ratio (*p* > 0.05); *, **, *** significant at *p* < 0.05, 0.01, and 0.001, respectively. ^‡^ Treatment means of the ANOVA test (values are the mean value of three replications). ^¥^ Values followed by the same letter, within the same row, were not significantly different (*p* < 0.05), Tukey’s multiple-range test.

**Table 4:** Total concentration of each chemical family of volatile compounds in smoked and non-smoked Iranian rice.

Compound	ANOVA ^†^	Non-Smoked Rice	Smoked Rice
Aldehydes	***	46.5 ± 3.6 a ^¥^	12.1 ± 1.2 b
Ketones	NS	1.19 ± 0.31 a	0.92 ± 0.25 a
Phenol derivatives	***	17.0 ± 3.8 b	44.9 ± 2.2 a
Furans	***	1.29 ± 0.24 b	35.6 ± 2.5 a
Linear hydrocarbons	***	33.3 ± 4.9 a	5.9 ± 1.6 b
Esters	***	0.12 ± 0.06 b	0.41 ± 0.16 a
Terpenes	*	0.60 ± 0.15 a	0.34 ± 0.06 b

^†^ NS = not significant F ratio (*p* > 0.05); *, *** significant at *p* < 0.05 and 0.001, respectively. ^‡^ Treatment means of the ANOVA test (values are the mean value of three replications). ^¥^ Values followed by the same letter, within the same row, were not significantly different (*p* < 0.05), Tukey’s multiple-range test.
